# Striatal-Enriched Protein Tyrosine Phosphatase Controls Responses to Aversive Stimuli: Implication for Ethanol Drinking

**DOI:** 10.1371/journal.pone.0127408

**Published:** 2015-05-20

**Authors:** Rémi Legastelois, Emmanuel Darcq, Scott A. Wegner, Paul J. Lombroso, Dorit Ron

**Affiliations:** 1 Department of Neurology, University of California San Francisco, San Francisco, California, United States of America; 2 Yale University School of Medicine, New Haven, Connecticut, United States of America; Mayo Clinic College of Medicine, UNITED STATES

## Abstract

The STriatal-Enriched protein tyrosine Phosphatase (STEP) is a brain-specific phosphatase whose dysregulation in expression and/or activity is associated with several neuropsychiatric disorders. We recently showed that long-term excessive consumption of ethanol induces a sustained inhibition of STEP activity in the dorsomedial striatum (DMS) of mice. We further showed that down-regulation of STEP expression in the DMS, and not in the adjacent dorsolateral striatum, increases ethanol intake, suggesting that the inactivation of STEP in the DMS contributes to the development of ethanol drinking behaviors. Here, we compared the consequence of global deletion of the *STEP* gene on voluntary ethanol intake to the consumption of an appetitive rewarding substance (saccharin) or an aversive solution (quinine or denatonium). Whereas saccharin intake was similar in STEP knockout (KO) and wild type (WT) littermate mice, the consumption of ethanol as well as quinine and denatonium was increased in STEP KO mice. These results suggested that the aversive taste of these substances was masked upon deletion of the *STEP* gene. We therefore hypothesized that STEP contributes to the physiological avoidance towards aversive stimuli. To further test this hypothesis, we measured the responses of STEP KO and WT mice to lithium-induced conditioned place aversion (CPA) and found that whereas WT mice developed lithium place aversion, STEP KO mice did not. In contrast, conditioned place preference (CPP) to ethanol was similar in both genotypes. Together, our results indicate that STEP contributes, at least in part, to the protection against the ingestion of aversive agents.

## Introduction

STriatal-Enriched protein tyrosine Phosphatase (STEP) is a phosphatase that is specifically expressed in the central nervous system (CNS) [1,2]. The *STEP* gene (*PTPN5*) produces alternatively spliced isoforms that include a 46 kDa cytosolic form (STEP_46_) and a 61 kDa membrane-associated form (STEP_61_) [3]. Both STEP_46_ and STEP_61_ have a wide distribution in the CNS, although STEP_61_ is enriched in striatum and to a lesser extent in lateral amygdala, hippocampus and cortex, while STEP_46_ is expressed in striatum and central nucleus of the amygdala [4]. STEP dephosphorylates and inactivates key neuronal signaling molecules including extracellular signal-regulated kinase1/2 (ERK1/2), stress-activated protein kinase p38 (p38), proline-rich tyrosine kinase 2 (Pyk2), Fyn kinase and the GluN2B subunit of the N-methyl-D-aspartate (NMDA) receptor [5,6,7,8,9,10,11]. As a consequence, STEP opposes the development of synaptic strengthening [11]. STEP is an important regulator of spatial [12] and fear conditioning learning processes [13], as well as motor skills learning and memory [14]. STEP rapidly inhibits p38 signaling after activation by NMDA receptors during learning processes and thereby prevents sustained neuronal excitation and functions as an important neuroprotector [8,15,16].

These studies demonstrate that STEP normally regulates several critical neurophysiological functions. In contrast, alterations of STEP expression and/or function contribute to several neurodegenerative diseases and psychiatric disorders that include Alzheimer’s disease (AD), Huntington’s chorea, and schizophrenia [11,17,18,19]. STEP was shown to be associated with physiological responses induced by cocaine [20], amphetamine [21] or ethanol [22], and STEP activity or expression is reduced after repeated and intermittent exposure to either stress [23] or ethanol [24]. We recently showed that the intermittent consumption of large amounts of ethanol induces a robust and long-lasting increase in the phosphorylation of STEP_61_ on a specific inhibitory site in the dorsomedial striatum (DMS) of mice, but not in other striatal regions [24]. Furthermore, we showed that knockdown of STEP_61_ specifically in the DMS increased ethanol intake and preference [24].

The development of ethanol drinking behaviors relies in part on the balance between the rewarding and aversive properties of ethanol [25,26]. As our recent data suggested that STEP_61_ inhibition was required for the development of ethanol consumption [24], here, we tested the hypothesis that STEP may modulate the intake of rewarding and/or aversive solutions. Therefore, we determined the consequences of global deletion of STEP on voluntary drinking of ethanol compared to voluntary consumption of sweet and bitter solutions.

## Materials and Methods

### Materials

Saccharin and quinine hemisulfate were purchased from Sigma (St Louis, MO). Denatonium benzoate was purchased from Alfa Aesar (Ward Hill, MA). Lithium chloride was purchased from Santa Cruz Biotechnology (Santa Cruz, CA).

### Ethics statement

All animal procedures in this report were approved by the University of California San Francisco (UCSF) Institutional Animal Care and Use Committee (AN091738-02G), and were conducted in agreement with the Guide for the Care and Use of Laboratory Animals and the Association for Assessment and Accreditation of Laboratory Animal Care (AAALAC, UCSF).

### Animals

Male and female STEP heterozygote mice were obtained from Jackson Laboratories. Pairs of male and female STEP heterozygote mice (C57BL/6 background) were mated in-house to generate STEP WT and KO littermates. Genotypes were determined by RT-PCR analysis of products derived from tail mRNA as described in [21].Male STEP WT and KO mice (2–4 months at the time of the experiments) were individually housed in a temperature- and humidity-controlled room under a 12-hr light/dark cycle, with food and water available *ad libitum*.

### Drugs and treatments

Ethanol solutions for the drinking experiments were prepared from absolute anhydrous ethanol (190 proof) diluted to 3–20% ethanol (*v/v*) in tap water. Saccharin, quinine hemisulfate, and denatonium benzoate were dissolved in tap water. For systemic administrations, lithium chloride was dissolved in saline and absolute anhydrous ethanol was diluted to 20% ethanol (*v/v*) in saline.

### Ethanol consumption

Oral ethanol intake was determined using continuous access to ethanol in a two-bottle choice drinking paradigm as previously described [27]. Briefly, drinking sessions were conducted 24 hrs a day, 7 days a week with one bottle containing tap water while the other contained an increasing concentration of ethanol (3, 6, 10 and 20%) with 7 days of access to each concentration. Fresh fluids were provided each time the concentration was changed. The bottles were weighed on days 2, 4, and 7 of each week and the mice were weighed once a week. The position (left or right) of each solution was alternated as a control for side preference.

### Saccharin, quinine and denatonium consumption

STEP WT and KO mice were tested for saccharin, quinine and denatonium intake using a continuous access two-bottle choice drinking paradigm. Drinking sessions were conducted 24 hrs a day, 7 days a week with one bottle containing tap water while the other contained an increasing concentration of saccharin (0.005, 0.015, 0.033 and 0.066%), quinine hemisulfate (0.01, 0.03, 0.06, 0.12 and 0.24mM), or denatonium benzoate (0.03, 0.06, 0.12 and 0.24 mM) with 4 days of access to each concentration. Fresh fluids were provided each time the solution was changed. The bottles were weighed every day and the mice were weighed once a week. The position (left or right) of each solution was alternated as a control for side preference.

### Conditioned place aversion

The conditioned place aversion procedure was performed according to [28]. The place conditioning boxes (Columbus Instrument) consist of two distinct compartments that differ in color and floor texture. After 5 days of handling and habituation to subcutaneous (s.c.) injections, the initial aversion of STEP WT and KO mice was assessed (preconditioning test). To do so, mice were allowed to freely explore both compartments for 20 min, and the time spent in each compartment was recorded. Three animals that spent more than 70% of the time in either one of the compartments during the preconditioning test were excluded from the study. Treatments were then further counterbalanced between compartments to use an unbiased procedure. The next day, the conditioning training started with two conditioning trials per day for 3 days. Specifically, mice were injected (s.c.) morning (9:00 am) and afternoon (4:00 pm) with either saline (vehicle-paired session) or 130 mg/kg lithium chloride (drug-paired session) and confined in the corresponding-paired compartment for 45 min. Control animals received saline injections mornings and afternoons followed by a 45 min confinement. On the fifth day (postconditioning test), mice had free access to both compartments for 20 min, and the time spent in each compartment was measured.

### Conditioned place preference

The conditioned place preference procedure was performed according to [29]. The place conditioning boxes were the same as used for the CPA experiment described above. After 5 days of handling and habituation to intraperitoneal (i.p.) injections, the initial preference of STEP WT and KO mice was assessed (preconditioning test). To do so, mice were allowed to freely explore both compartments for 30 min, and the time spent in each compartment was recorded. One animal that spent more than 70% of the time in either one of the compartments during the preconditioning test was excluded from the study. Treatments were counterbalanced between compartments to use an unbiased procedure. The next day, the conditioning training started with one conditioning trial per day for 8 days. Specifically, mice were administered (i.p.) saline solution and confined in the vehicle-paired compartment for 5 min. The next day, mice were administered (i.p.) ethanol solution (2.0 g/kg) and confined in the ethanol-paired compartment for 5 min. Control animals received saline injections instead of ethanol injections. This schedule was repeated three more times (i.e., for 4 saline- and 4 ethanol-conditioning trials). On the tenth day (postconditioning test), mice had free access to both compartments for 30 min, and the time spent in each compartment was measured.

### Locomotor activity

Spontaneous locomotor activity of mice was measured in activity monitoring chambers (43 cm × 43 cm) with horizontal photo beams (Med Associates, St Albans, VT). Horizontal locomotor activity was monitored and the distance traveled (cm) by the mice was recorded for 30 min.

### Statistical analysis

Data was analyzed with two-way analysis of variance (ANOVA) or two-way repeated measures-ANOVA (RM-ANOVA). Significant main effects and interactions of the ANOVAs were further investigated with the Student-Newman-Keuls (SNK) *post hoc* test or method of contrast analysis. Statistical significance was set at *p* < 0.05.

## Results

### STEP controls the consumption of ethanol, quinine and denatonium, but not the consumption of saccharin

**Fig 1:**
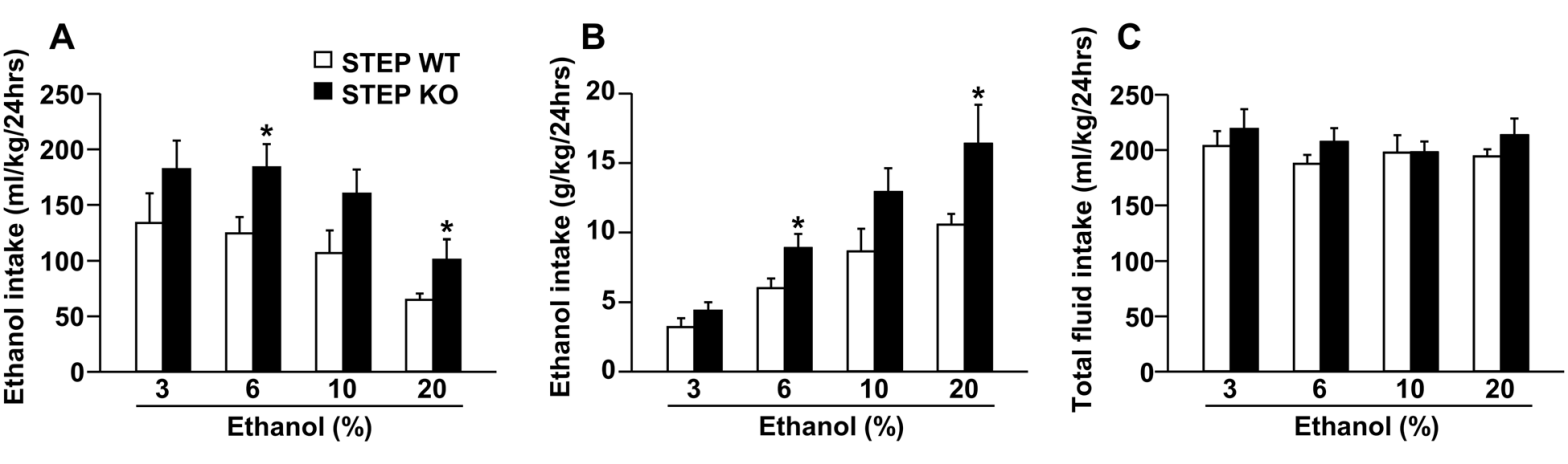
Global deletion of STEP increases ethanol consumption. STEP WT and KO mice were submitted to a continuous access two-bottle choice paradigm with access to one bottle of an ethanol solution and one bottle of tap water. Ethanol concentration was increased every week (3, 6, 10 and 20%) with 7 days of access to each concentration. Results are expressed as mean ± SEM of ethanol consumed expressed in (**A**) ml of solution and (**B**) g of pure ethanol per kg of body weight as well as (**C**) total fluid intake per 24 hours for each ethanol concentration. Two-way RM-ANOVA showed an effect of genotype for **A** [*F*(1,12) = 4.969, *p* = .046], **B** [*F*(1,12) = 5.164, *p* = .042] but not for **C** [*F*(1,12) = .954, *p* = .348], an effect of ethanol concentration for **A** [*F*(3,36) = 15.565, *p* <.001], **B** [*F*(3,36) = 42.103, *p* <.001] but not for **C** [*F*(3,36) = .959, *p* = .423] and no interaction between genotype and ethanol concentration for **A** [*F*(3,36) = 0.261, *p* = .853], **B** [*F*(3,36) = 2.358, *p* = .088] and **C** [*F*(3,36) = .469, *p* = .706]. *p <.05 vs. WT, method of contrasts. n = 6–8.

We recently showed that the inhibition of STEP_61_ in mice DMS is required for the development of ethanol-drinking behaviors [24]. Specifically, we showed that the voluntary consumption of ethanol induces a robust inhibition of STEP_61_ in the DMS of mice and that knockdown of STEP_61_ in the DMS increased ethanol intake [24]. Consumption is strongly correlated with the rewarding properties of ethanol [30]. However, ethanol intake in both rodents [31] and humans [32,33] is also tempered by their sensitivity to the aversive bitter taste of ethanol. Therefore, we tested whether global deletion of the *STEP* gene in mice leads to changes in the consumption of ethanol (rewarding and bitter [34]), saccharin (rewarding) and quinine and denatonium (aversive) solutions. To do so, STEP WT and KO mice underwent a continuous access to ethanol in a two-bottle choice procedure, during which ethanol concentration was increased every week (from 3% to 20%). Similar to knockdown of STEP_61_ in the DMS [24], STEP KO mice consumed more ethanol compared to their WT littermates (Fig 1A and 1B), whereas total fluid intake remained unchanged (Fig 1C), suggesting that STEP controls ethanol consumption.

**Fig 2:**
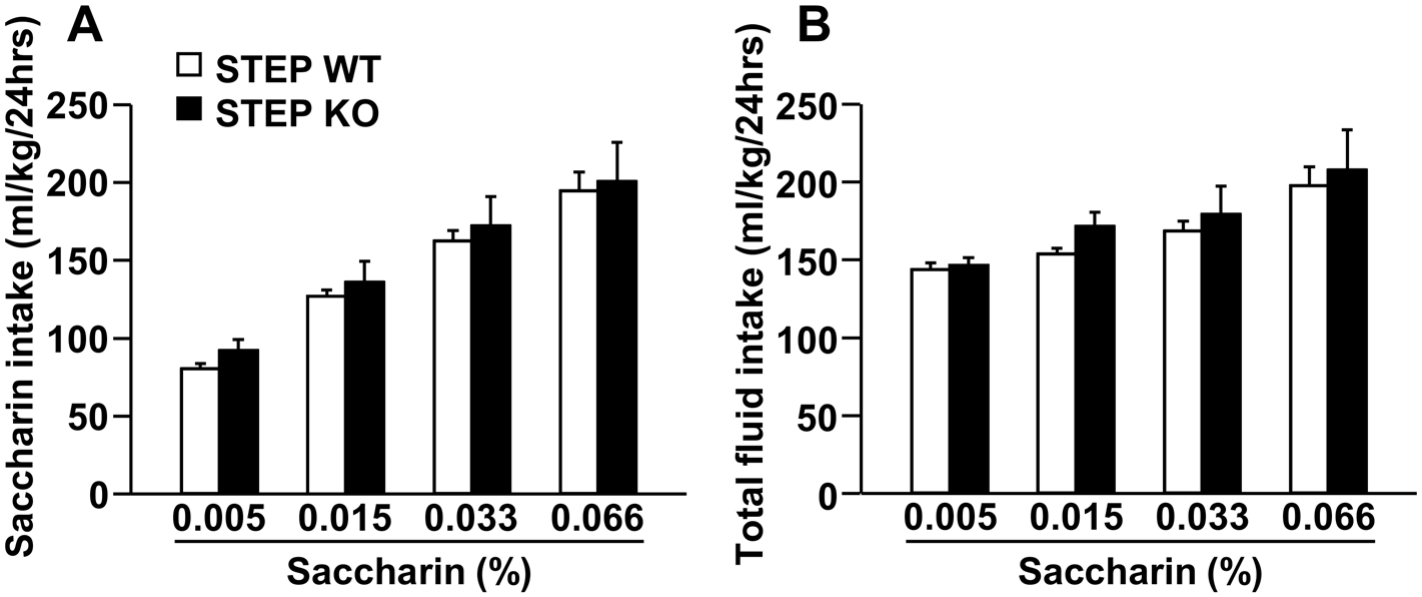
Saccharin consumption is similar in STEP KO and WT mice. STEP WT and KO mice were submitted to a continuous access two-bottle choice paradigm with access to one bottle of a saccharin solution and one bottle of tap water. Saccharin concentration was increased every 4 days (0.005, 0.015, 0.033 and 0.066%). Results are expressed as mean ± SEM of (**A**) saccharin or (**B**) total fluid intake per 24 hours for each saccharin concentration. Two-way RM-ANOVA showed no effect of genotype for **A** [*F*(1,15) = .357, *p* = .559] and **B** [*F*(1,15) = .508, *p* = .487], an effect of saccharin concentration for **A** [*F*(3,45) = 72.3, *p* <.001] and **B** [*F*(3,45) = 17.207, *p* <.001] and no interaction between genotype and saccharin concentration for **A** [*F*(3,45) = .0441, *p* = .988] and **B** [*F*(3,45) = .284, *p* = .837]. n = 8-9.

**Fig 3:**
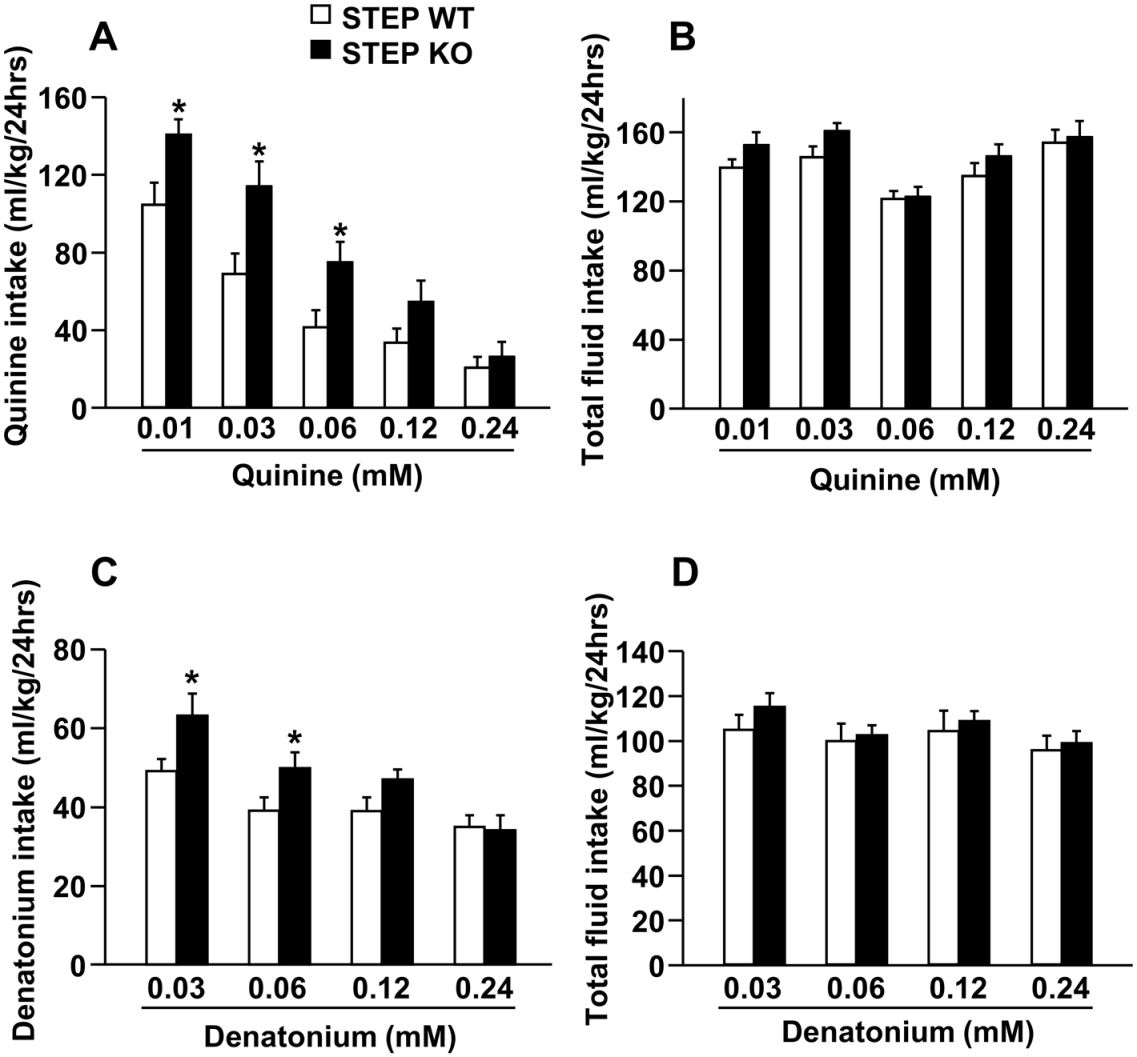
Quinine and denatonium consumption is increased in STEP KO vs. WT mice. STEP WT and KO mice were submitted to a continuous access two-bottle choice paradigm with access to one bottle of a quinine or denatonium solution and one bottle of tap water. Quinine or denatonium concentration was increased every 4 days (0.01, 0.03, 0.06, 0.12 and 0.24 mM for quinine and 0.03, 0.06, 0.12 and 0.24 mM for denatonium). Results are expressed as mean ± SEM of (**A**) quinine or (**C**) denatonium intake or total fluid intake per 24 hours for each (**B**) quinine or (**D**) denatonium concentration. Two-way RM-ANOVA showed an effect of genotype for **A** [*F*(1,15) = 7.524, *p* = .015] and **C** [*F*(1,16) = 4.594, *p* = .048] but not for **B** [*F*(1,15) = 1.181, *p* = .294] and **D** [*F*(1,16) = 0.365, *p* = .554], an effect of concentration for **A** [*F*(4,60) = 64.307 *p* <.001], **B** [*F*(4,60) = 24.150, *p* <.001], **C** [*F*(3,48) = 19.434, *p* <.001] and **D** [*F*(3,48) = 10.080, *p* <.001], and no interaction between genotype and concentration for **A** [*F*(4,60) = 2.435, *p* = .057], **B** [*F*(4,60) = 1.290, *p* = .284], **C** [*F*(3,48) = 2.462, *p* = .074] and **D** [*F*(3,48) = 1.007, *p* = .398]. *p <.05 vs. WT, method of contrasts. n = 8–9.

Next, we tested the consumption of saccharin and quinine solutions in STEP WT and KO mice in a continuous access two-bottle choice procedure, with the concentration of saccharin (0.005% to 0.066%) or quinine (0.01 mM to 0.24 mM) increasing every four days. As shown in Fig 2A and 2B, saccharin intake, as well as total fluid intake, was similar in both genotypes at all saccharin concentrations. On the other hand, we found that deletion of the STEP gene disrupted quinine consumption. Specifically, quinine intake was significantly increased at three out of four of quinine concentrations (i.e. 0.01, 0.03 and 0.06 mM) in STEP KO mice compared to WT littermate mice (Fig 3A). Importantly, total fluid intake was similar between both genotypes (Fig 3B). We next tested the drinking of another bitter substance with an unrelated structure, denatonium, in STEP WT and KO mice using a continuous access two-bottle choice procedure, with the concentration of denatonium increased every four days (0.03 mM to 0.24 mM). We found that STEP KO mice drank more denatonium than their WT littermate mice at the denatonium concentrations of 0.03 mM and 0.06 mM (Fig 3C), whereas total fluid intake was unaltered (Fig 3D).

**Fig 4:**
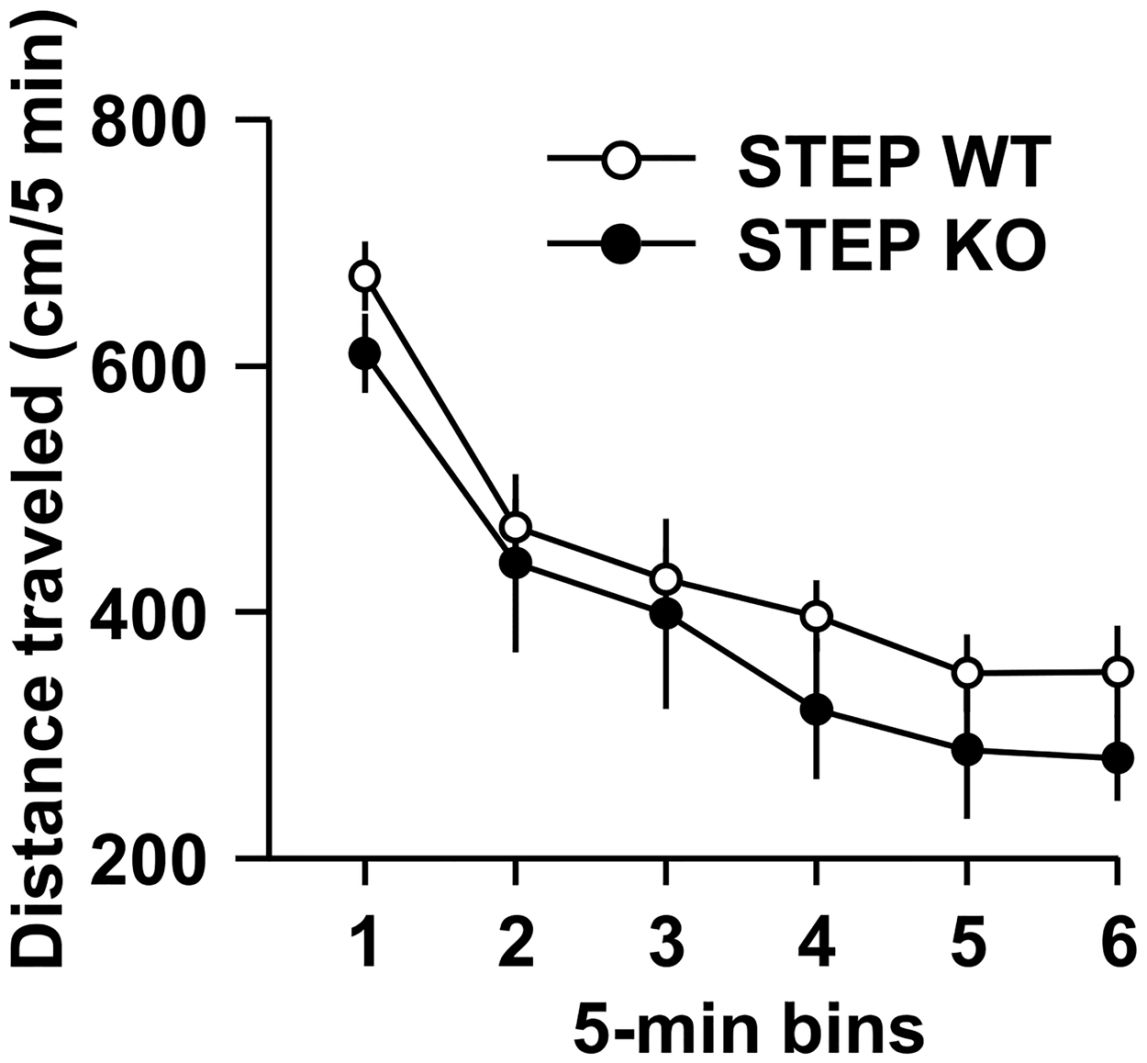
Global deletion of STEP does not alter spontaneous locomotor activity. Results are expressed as mean ± SEM of distance traveled (cm) per 5-min bins in STEP WT and KO mice during a 30-min session. Two-way RM-ANOVA showed an effect of time [*F*(5,60) = 49.091, *p* <.001] but no effect genotype [*F*(1,12) = 1.161, *p* = .302] and no interaction between time and genotype [*F*(5,60) = .355, *p* = .877]. n = 6–8.

We next determined whether the increase in ethanol, quinine and denatonium intake upon deletion of the *STEP* gene was due to alteration in spontaneous locomotor activity. As shown in Fig 4, the distance traveled in an open field was unaltered in STEP KO mice compared to WT mice, indicating that deletion of STEP does not change spontaneous locomotion. Thus, the observed increase in the ingestion of aversive tasting agents such as quinine, denatonium and ethanol is not due to a general increase of spontaneous locomotion. Together, these results suggest that STEP controls the ingestion of aversive tasting agents such as quinine, denatonium and ethanol.

### STEP contributes to conditioned place aversion to lithium chloride

**Fig 5:**
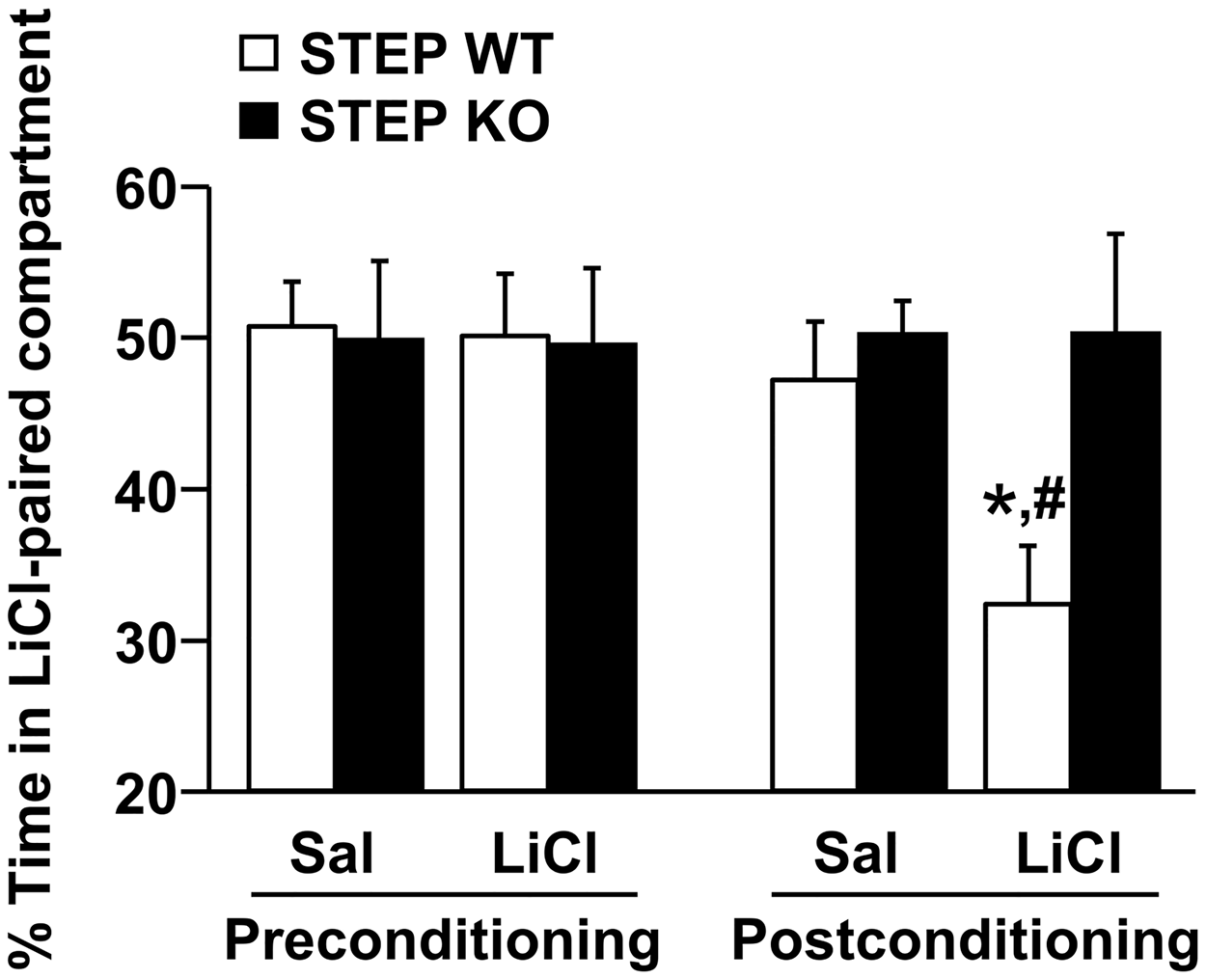
STEP KO mice do not express conditioned place aversion to lithium chloride. STEP WT and KO mice were submitted to a conditioned place aversion paradigm. The conditioning phase consisted in 3 days of daily administration (s.c.) of saline (mornings) or 130 mg/kg lithium chloride (LiCl; afternoons) solution followed by the confinement in the saline- or LiCl-paired compartment for 45 min (3 saline and 3 LiCl conditioning sessions). One day after the third conditioning day, a 20-min postconditioning test was conducted. Results are expressed as mean ± SEM of the percentage of time spent in the LiCl-paired compartment during the preconditioning and postconditioning tests. Two-way ANOVA conducted on the postconditioning values showed an effect of genotype [*F*(1,28) = 4.784, *p* = .037], no effect of conditioning [*F*(1,28) = 2.395, *p =* .133] and no interaction between genotype and conditioning [*F*(1,28) = 2.460, *p* = .128]. *p <.05 vs. Saline, ^#^ *p* <.05 vs. KO, method of contrasts. n = 7–9.

Our data thus far suggest that the increase in ethanol intake upon *STEP* gene deletion arises, at least in part, from an increase in the threshold of bitter taste rejection. As bitter taste is an aversive stimulus that often signals harmful poisonous substances and is generally avoided by mammals [35,36], we thought to assess whether STEP is involved in the avoidance toward aversive stimuli. To do so, we submitted STEP WT and KO mice to a conditioned place aversion (CPA) paradigm using lithium chloride a substance that when administered systemically causes gastric malaise similar to food poisoning [37]. The procedure consisted of three conditioning sessions to saline and three conditioning sessions to 130 mg/kg lithium chloride as previously described [28]. Whereas WT mice developed a strong aversion to the lithium-paired compartment, STEP KO mice failed to express any avoidance to the environment associated with the aversive stimulus (Fig 5). These results suggest that STEP contributes to the avoidance to aversive stimuli in mice.

### Conditioned place preference to ethanol is not altered in STEP KO mice

**Fig 6:**
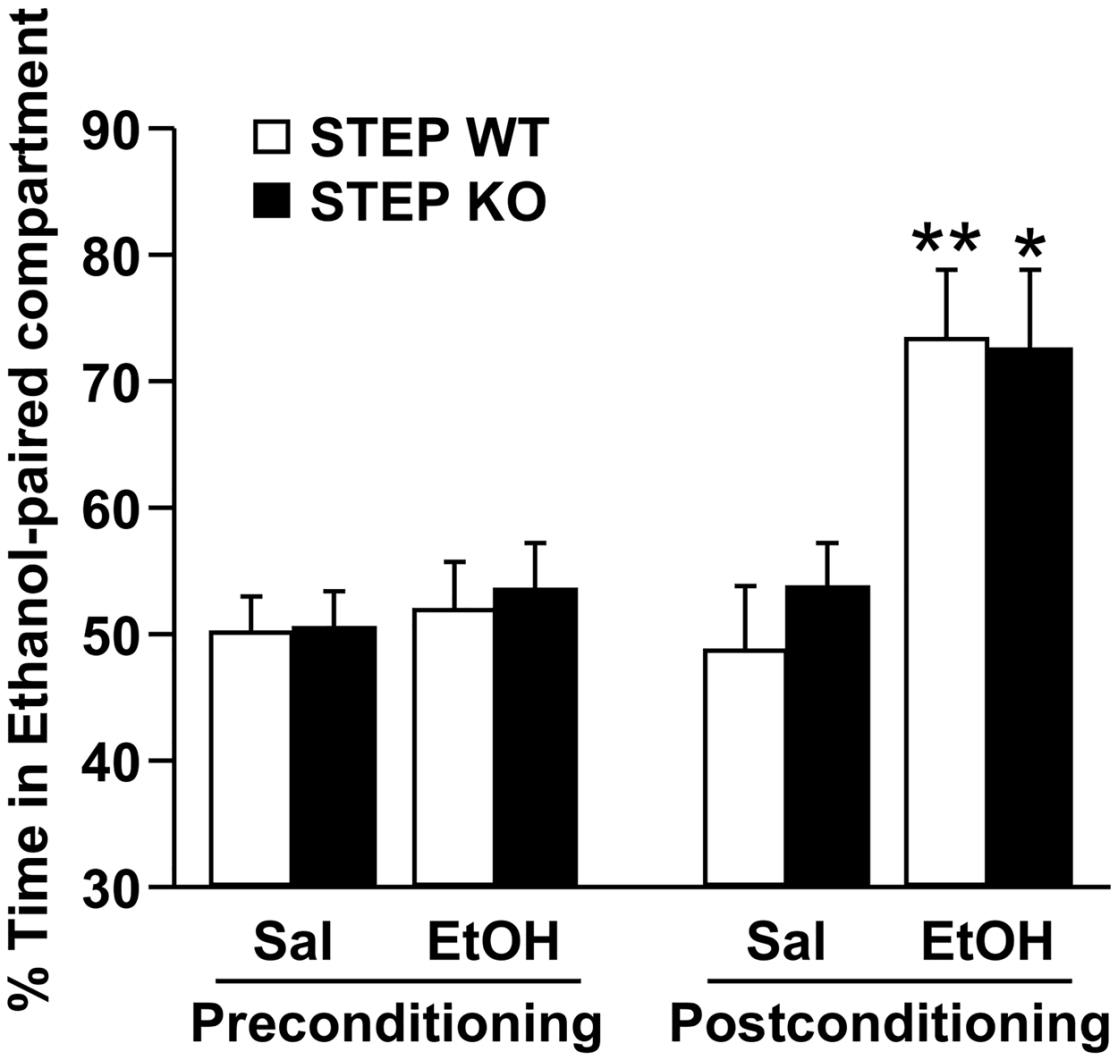
Conditioned place preference to ethanol is not altered in STEP KO mice. STEP WT and KO mice were submitted to an ethanol-induced conditioned place preference paradigm. The conditioning phase consisted in 8 days of daily administration (i.p.) of either saline (Sal) or 2.0 g/kg ethanol (EtOH) solution followed by the confinement in the saline- or ethanol-paired compartment for 5 min (4 saline and 4 ethanol conditioning sessions). One day after the eighth session, a 30-min postconditioning test was conducted. Results are expressed as mean ± SEM of the percentage of time spent in the ethanol-paired compartment during the preconditioning and postconditioning tests. Two-way ANOVA conducted on the postconditioning values showed no effect of genotype [*F*(1,33) = .168, *p* = .684], an effect of conditioning [*F*(1,33) = 18.587, *p <*.001.] and no interaction between genotype and conditioning [*F*(1,33) = .330, *p* = .570.]. *p <.05, **p <.01 vs. Saline, method of contrasts. n = 8–10.

As ethanol has a bitter taste component [34], we thought that this mechanism could explain, at least in part, the increase of ethanol consumption in STEP KO mice. We therefore tested whether STEP signaling also controls the sensitivity to the pharmacological rewarding properties of ethanol. We used an ethanol-induced conditioned place preference (CPP), which measures the rewarding properties of drugs of abuse [38], in STEP WT and KO mice. The procedure consisted of four conditioning sessions to saline and four conditioning sessions to 2.0 g/kg ethanol as previously described [29]. STEP WT and KO mice expressed a similar CPP to ethanol (Fig 6), suggesting that, under these conditions, STEP does not control the response to ethanol’s pharmacological rewarding properties.

## Discussion

We report here that global STEP_61_/STEP_46_ deletion increased ethanol consumption in mice. We also showed that quinine and denatonium consumption was increased in STEP KO mice compared to WT littermates, whereas saccharin and total fluid intake as well as spontaneous locomotion were unaltered. Our results strongly suggest that STEP controls the consumption of solutions with a bitter taste component such as ethanol, quinine and denatonium. We further showed that mice with global deletion of the *STEP* gene did not develop aversion to the gastric malaise-inducer lithium chloride, although they expressed similar levels of conditioned place preference for ethanol compared to their WT littermates, indicating that STEP plays an important role in the avoidance to aversive stimuli in mice. Altogether, our results suggest a novel mechanism by which STEP participates to the protection against the ingestion of aversive tasting agents like ethanol.

Using a continuous access two-bottle choice of increasing concentration of ethanol, we showed that STEP KO mice drank significantly more ethanol than WT mice, in line with our recent findings [24]. Importantly, it has been shown that ethanol clearance is similar between STEP WT and KO mice [22], ruling out the possibility that the increase in ethanol drinking upon *STEP* gene deletion could be attributable to an enhanced ethanol metabolism, nor was it due to locomotor changes, as spontaneous locomotor activity was similar between both genotypes. Besides the sensitivity to the rewarding properties of ethanol, the perception of its aversive bitter flavor [34,39] also plays an important role in the levels of voluntary oral consumption of ethanol in both rodents [31] and humans [32,33]. We therefore hypothesized that STEP may promote the avoidance response to solutions with an aversive bitter taste. As STEP KO were aversion-resistant compared to WT littermate mice, our results suggest that STEP is specifically involved in the avoidance to the bitter tastant ethanol. Consistent with our hypothesis, a relationship between the responsiveness to quinine and ethanol has been suggested [40], and a blunted sensitivity to bitter taste may be an important predictor of high ethanol consumption in both rodents [31] and humans [32,33].

To further confirm that STEP contributes to the avoidance to bitter substances, we tested the drinking of another structurally unrelated bitter substance, denatonium, in STEP WT and KO mice. Even though taste receptor cells discriminate between different bitter stimuli [41], rats fail to distinguish quinine from denatonium [42]. Here we showed that STEP KO mice drank more denatonium than WT mice, confirming the involvement of STEP in the avoidance to bitter tastants.

Bitter taste often signals harmful substances and sensitivity to bitter tastants is thus acknowledged to have evolved as a protective mechanism in mammals [35,36]. Therefore, a bitter taste is considered as an aversive stimulus in rodents [43]. To test whether STEP contributes to the avoidance to aversive stimuli in mice, we used a lithium chloride-induced CPA paradigm. We choose lithium chloride as the aversive stimulus in the CPA experiment because the gastric malaise induced by lithium chloride administration is comparable to food poisoning [37], which can be the result of the consumption of bitter substances [35]. We found that WT mice developed a robust CPA whereas STEP KO mice were resistant to CPA. The fact that comparable ethanol-induced CPP levels were observed between STEP WT and KO mice allowed us to rule out spatial or associative learning deficits as confounding factors in the CPA experiment. These results indicate that STEP is necessary for the development of conditioned aversion and further suggest that inactivation of STEP abolishes the avoidance to aversive stimuli and hence promotes the consumption of aversive tasting agents like ethanol.

We recently demonstrated that inactive STEP_61_ in the DMS increases the drinking of ethanol without altering the palatability of sweet or bitter solutions [24], in apparent contrast with the present report. First, we cannot exclude the occurrence of developmental compensatory mechanism in the mutant mice used herein compared to the gene knock down acutely performed in our previous study. Such compensation may account, at least in part, for the behavioral changes observed in the STEP KO mutant mice. Moreover, it is important to note that herein, both STEP_61_ and STEP_46_ isoforms are deleted in the whole brain, versus specific downregulation of the 61 kDa isoform in the DMS in our previous study [24]. Thus, ethanol-drinking behaviors are driven by different STEP-related mechanisms in several discrete brain regions, some of them regulating the appetence toward the pharmacological effects of ethanol in the DMS and others controlling the aversion to the bitter taste of ethanol. For instance, STEP is expressed in brain regions such as central amygdala and bed nucleus of the stria terminalis [23], both of which are involved in negative affective learning [44,45] and responsive to bitter tastants [46,47]. Further studies will thus be necessary to dissect the contribution of STEP to aversive stimuli in such brain regions.

Recent preclinical reports indicate that STEP inactivation improves cognitive deficits in animal models of AD [19,48], Hence, STEP inhibitors are currently being developed as potential treatments for humans suffering from AD [11,49]. Our previous study [24] and the present report indicate that both specific downregulation of STEP_61_ expression in the DMS and global deletion of the *STEP* gene lead to the increase of ethanol drinking. Therefore, future use of STEP inhibitors in clinical trials should be closely monitored regarding the consumption of alcoholic beverages and the response to aversive, undesirable signals.
